# Some Observations on the Gout, from an Unpublished Paper on That Subject, by a Celebrated Physician Lately Deceased
*This paper, for the heads of which we are indebted to a very respectable correspondent, seems the more interesting as it was written only a short time before the author's death, and of course may be considered as one of the latest, if not the last production of his pen on a medical subject.


**Published:** 1781-03

**Authors:** 


II. *Some Obfervations on the Gout, from an unpublifhed Paper* on that Subject, by a celebrated phyfician lately deceafed.*

THE author fets out with obferving, that in the regular gout, the fharper the pain is, the fhorter will be the fit. He condemns the ufe of wine and the high living that are fo generally adopted with a view to affift nature in throwing off the gouty matter. He has fometimes

* This paper, for the heads of which we are indebted to a very refpectable correfpondent, feems the more interefting as it was written only a fhort time before the author's death, and of courfe may be confidered as one of the lateft, if not the laft production of his pen on a medical fubject.

ſometimes ſeen a doſe of the tinctura thebaica of uſe; but when frequently repeated it checked the progreſs of the fit, the criſis was of courſe leſs perfect, and its bad effects on the conſtitution ſoon became evident.

He likewiſe thinks the wrapping up the gouty limb in too much flannel hurtful; they who do ſo being conſtantly obſerved to recover the tone of it ſlowly. He obſerves, that a free admiſſion of cold air would probably be equally improper. He has found a moderate warmth to be the beſt, ſuch as keeps up a proper degree of action in the part without being too ſtimulating.

Rye meal applied in the way of poultice to the gouty limb, was a favourite application with the late Dr. Gowin Knight; but the author has ſeen it pernicious even in Dr. Knight's own caſe. He inſtances the caſe of a gentleman, one of his patients, who by flouring his ſtocking, repelled the gout from his foot, and threw it into his knee. A ſimilar application had the effect of driving it back again into his foot.

He has ſeen compreſſion on the foot fix a wandering gout. In his own caſe, upon feeling irregular gouty pains, he ordered a pair of ſhoes

ſhoes that were too tight for him to be aired, and having put them on, in leſs than half an hour he felt the gout in his foot.

He obſerves, that ſudden repulſions of the gout, by external applications, produce various diſeaſes. He once had occaſion to ſee a genuine phthiſis pulmonalis in a man of forty years of age, which evidently aroſe from this cauſe.

Speaking of Le Fevre's medicine for the gout, our author obſerves, that Le Fevre was ſo extremely cautious in the exhibition of his ſpecific, that he placed it upon the patient's tongue himſelf, and took care to ſee them ſwallow it. He has made inquiry of ſeveral of Le Fevre's patients, who all agreed that it had no other taſte than that of ſugar.

Upon looking into the obſervations of a German ſurgeon who practiſed at Aſtrachan, and whoſe work our author cauſed to be tranſlated into Engliſh for his own private ſatisfaction, he found a medicine recommended for the cure of the gout, the principal, and indeed the only active ingredient of which is white vitriol, given in the doſe of one grain. Our author, therefore, very juſtly ſuſpects, that Le Fevre's medicine was nothing more

 than than white vitriol mixed with ſugar, and that as this might have been eaſily detected, his cautious mode of proceeding may be accounted for from this circumſtance.

In treating of the irregular gout, our author diſtinguiſhes the ſpaſm and pain that are frequently produced by acrid bile, from the gout. In the bilious complaint, the pain is violent about the præcordia, and oftentimes attended with ſpaſm of the neighbouring parts. On the other hand, in the gout, the pain is uſually moſt acute about the tip of the cartilago enſiformis.

Our author obſerves, that keeping the body open will be uſeful; but he condemns violent evacuations of any kind in the gout. Bliſtering near the ſeat of the complaint, he thinks may be improper, both in this and in ſome other diſeaſes. Thus he remarks, for inſtance, that when the gout affects the head, bliſters will do beſt applied to the feet; and that in delirium in fevers the ſame rule will hold good, becauſe bliſters to the head may occaſion too great an afflux to that part of the body.

